# Excavatolide B Attenuates Rheumatoid Arthritis through the Inhibition of Osteoclastogenesis

**DOI:** 10.3390/md15010009

**Published:** 2017-01-06

**Authors:** Yen-You Lin, Yen-Hsuan Jean, Hsin-Pai Lee, Sung-Chun Lin, Chieh-Yu Pan, Wu-Fu Chen, Shu-Fen Wu, Jui-Hsin Su, Kuan-Hao Tsui, Jyh-Horng Sheu, Ping-Jyun Sung, Zhi-Hong Wen

**Affiliations:** 1 Department of Marine Biotechnology and Resources, National Sun Yat-sen University, No.70, Lianhai Road, Gushan District, Kaohsiung 80424, Taiwan; chas6119@gmail.com (Y.-Y.L.); ma4949@adm.cgmh.org.tw (W.-F.C.); sheu@mail.nsysu.edu.tw (J.-H.S.); 2 Department of Orthopaedic Surgery, Ping-Tung Christian Hospital, No.60, Dalian Road, Pingtung 90059, Taiwan; jean.tang@msa.hinet.net (Y.-H.J.); hplee0929@gmail.com (H.-P.L.); linsungchun@yahoo.com.tw (S.-C.L.); 3 Department and Graduate Institute of Aquaculture, National Kaohsiung Marine University, No.142, Haizhuan Road, Nanzi District, Kaohsiung 81157, Taiwan; panjade@webmail.nkmu.edu.tw; 4 Department of Neurosurgery, Chang Gung Memorial Hospital-Kaohsiung Medical Center and Chang Gung University College of Medicine, No.123, Dapi Road, Niaosong District, Kaohsiung 83301, Taiwan; 5 Department of Neurosurgery, Xiamen Chang Gung Memorial Hospital, No.123, Xiafei Road, Haicang District, Xiamen 361000, China; 6 Department of Life Science, Institute of Molecular Biology, National Chung-Cheng University, No.168, Sec. 1, University Road, Min-Hsiung, Chia-yi 62102, Taiwan; biosfw@ccu.edu.tw; 7 Taiwan Coral Research Center, National Museum of Marine Biology & Aquarium, No.2 Houwan Road, Checheng, Pingtung 94450, Taiwan; x2219@nmmba.gov.tw (J.-H.S.); pjsung@nmmba.gov.tw (P.-J.S.); 8 Graduate Institute of Marine Biotechnology, National Dong Hwa University, No.2 Houwan Road, Checheng, Pingtung 94450, Taiwan; 9 Department of Obstetrics and Gynecology, Kaohsiung Veterans General Hospital, No.386, Dazhong 1st Road, Zuoying District, Kaohsiung 81362, Taiwan; khtsui60@gmail.com; 10 Department of Obstetrics and Gynecology and Institute of Clinical Medicine, National Yang-Ming University, No.155, Sec. 2, Linong Street, Taipei 11221, Taiwan; 11 Department of Pharmacy and Graduate Institute of Pharmaceutical Technology, Tajen University, No.20, Weixin Road, Yanpu, Pingtung 90741, Taiwan; 12 Doctoral Degree Program in Marine Biotechnology, National Sun Yat-sen University and Academia Sinica, No.70, Lianhai Road, Gushan District, Kaohsiung 80424, Taiwan

**Keywords:** osteoclast, osteoclastogenesis, rheumatoid arthritis (RA), excavatolide B, (Exc-B)

## Abstract

Osteoclasts are multinucleated giant cells of macrophage/monocyte lineage, and cell differentiation with the upregulation of osteoclast-related proteins is believed to play a major role in the destruction of the joints in the course of rheumatoid arthritis (RA). Pro-inflammatory cytokines, such as interleukin-17A (IL-17A) and macrophage colony-stimulating factor (M-CSF), can be overexpressed in RA and lead to osteoclastogenesis. In a previous study, we found that cultured-type soft coral-derived excavatolide B (Exc-B) exhibited anti-inflammatory properties. In the present study, we thus aimed to evaluate the anti-arthritic activity of Exc-B in in vitro and in vivo models. The results demonstrated that Exc-B inhibits LPS-induced multinucleated cell and actin ring formation, as well as TRAP, MMP-9, and cathepsin K expression. Additionally, Exc-B significantly attenuated the characteristics of RA in adjuvant (AIA) and type II collagen-induced arthritis (CIA) in rats. Moreover, Exc-B improved histopathological features, and reduced the number of TRAP-positive multinucleated cells in the in vivo AIA and CIA models. Immunohistochemical analysis showed that Exc-B attenuated the protein expression of cathepsin K, MMP-2, MMP-9, CD11b, and NFATc1 in ankle tissues of AIA and CIA rats. Level of interleukin-17A and macrophage colony-stimulating factor were also decreased by Exc-B. These findings strongly suggest that Exc-B could be of potential use as a therapeutic agent by inhibiting osteoclast differentiation in arthritis. Moreover, this study also illustrates the use of the anti-inflammatory marine compound, Exc-B, as a potential therapeutic strategy for RA.

## 1. Introduction

Rheumatoid arthritis (RA) is an autoimmune-mediated inflammatory disease characterized by synovial inflammation, as well as joint damage, cartilage destruction, and bone erosion with osteoclast activation [1]. Epidemiology studies indicate that RA could result from the combined effects of environmental and genetic risk factors [2]. Presently, the disease affects approximately 1% of the adult population worldwide, and is three times more likely to occur in women than in men [3]. Typically, the immune system will attack the joint tissue, leading to redness, oedema, and ankyloses [4,5]. Invasion of the pannus by large clusters of neutrophils, macrophages, lymphocytes, mast cells, and multinucleated cells is a typical histopathological feature of joint destruction [6,7]. Pannus formation leads to an increase in the number of blood vessels by angiogenesis and greater immune cells clustering and infiltration in the synovial tissue [8]. The pannus can cover the cartilage and bone surface and cause joint damage involving complex immune and inflammatory responses by different infiltrating cells [8]. The up-regulation of pro-inflammatory cytokines, proteases, and matrix metalloproteinases, released from infiltrating cells of the pannus, can induce changes in chondrocyte metabolism and matrix degradation, leading to cartilage damage and bone erosion [9]. Current treatment strategies for RA include non-steroidal anti-inflammatory drugs (NSAIDs), corticosteroids, disease-modifying anti-rheumatic drugs (DMARDS), and biologic response modifiers [3]. Moreover, for more effective treatment of RA, antagonists of tumour necrosis factor alpha (TNF-α), interleukin 1 (IL-1), interleukin 6 (IL-6), and interleukin 17A (IL-17A) are also considered in treatment strategies, as these cytokines play important roles in the inflammatory process in RA [3].

Invasion and destruction of the joint, in the progression of RA, is mediated by osteoclast differentiation and the upregulation of osteoclast-related proteins [6,10]. Osteoclasts are multinucleated cells, which are mainly differentiated from bone marrow hematopoietic monocyte/macrophage lineages [7,11]. In previous studies, inflammatory cells such T lymphocytes, macrophages, neutrophils, as well as other cell types have been shown to release pro-inflammatory proteins, such as TNF-α, IL-17A, and macrophage colony-stimulating factor (M-CSF), and promote clusters of differentiation molecule 11b (CD11b) positive cells, leading to osteoclastogenesis in RA [1,12,13,14]. Previous studies indicated that CD11b is a cell marker for macrophages [15]. In osteoclastogenesis, multinuclear osteoclasts are derived from CD11b-positive mononuclear cells and CD11b is considered a cell surface marker of osteoclast precursors, which were induced by M-CSF and RANKL, leading to osteoclast formation [13,14]. Previous studies indicated that the up-regulation of inflammatory cytokine IL-17A, which is mainly secreted by T helper 17 cells (Th17 cells), also plays an important role in promoting RA progression [16,17,18]. Th17 cells trigger infiltrating cells to release more pro-inflammatory mediators, such as TNF-α, MMPs, IL-1, and nitric oxide (NO) through IL-17A stimulation, thereby causing RA [19]. Moreover, IL-17A also promotes ostoeclastogenesis by stimulating osteoblasts to release RANKL [19]. These pro-inflammatory mediators lead to upregulation of the nuclear factor of activated T cells, cytoplasmic 1 (NFATc1)-related pathway to form multinucleated cells in RA. NFATc1 is a key transcription factor for upregulating the osteoclast-related protein expressions of cathepsin K, matrix metalloproteinase-9 (MMP-9), and tartrate-resistant acid phosphatase (TRAP), and promoting cell fusion. Subsequently, osteoclast differentiation results in bone resorption through activation of the nuclear factor-κB ligand (RANKL)/receptor activator of nuclear factor-κB (RANK)/osteoprotegerin (OPG) pathway in osteoclastogenesis [20,21]. Previous studies also indicate that anti-inflammatory compounds, such as celastrol and saponin, significantly attenuate RA via the downregulation of osteoclast differentiation. Thus, we propose that a marine-derived anti-inflammatory compound could also have potential use in the treatment of RA.

A vast number of studies has demonstrated the use of lipopolysaccharide (LPS), which is present in the membranes of Gram-negative bacteria-challenged RAW 264.7 cells, as a well-established model for in vitro anti-inflammatory screening [22,23]. Previous research also indicates that LPS could induce the formation of multinucleated cells with expressed TRAP protein via the nuclear factor-kappa B (NF-κB) and mitogen-activated protein kinases (MAPKs) pathways [24]. On the other hand, adjuvant-induced arthritis (AIA) and type II collagen-induced arthritis (CIA) are also widely used animal models for the study of RA, and for evaluating the therapeutic potential of anti-RA agents [25,26,27]. High levels of pro-inflammatory cytokines and TRAP-positive multinucleated cells that drive RA progression have also been detected in animal models [28,29,30]. Thus, we sought to examine the effect of excavatolide B (Exc-B) on LPS-induced osteoclastogenesis in murine macrophage RAW264 cell in the present study.

Recently, soft corals have been recognized as an important resource for drug development, and nearly 3000 compounds have been discovered in corals over the past two decades [31,32]. In secondary metabolites isolated from soft corals, several potential candidate compounds with anti-inflammatory activity have been identified [33]. For instance, previous research shows the anti-inflammatory potential of briarane-type diterpene, which is isolated from Taiwanese gorgonian coral [34,35]. In this study, we aimed to examine the effects of the anti-inflammatory briarane-type diterpene, Exc-B, and the possible mechanisms involved in its amelioration of RA progression. Exc-B was first isolated from the wild-type gorgonian coral *Briareum excavatum*, and has been shown to exhibit relatively low cytotoxicity [36]. In a previous study, Wei et al. showed the anti-inflammatory and immunodulatory effects of Exc-B in 12-*O*-Tetradecanoylphorbol-13-acetate (TPA) induced inflammation and dermatitis in murine skin, as well as in LPS-induced mouse bone marrow-derived dendritic cells, by reduction of IL-6 and TNF-α protein expression [37]. Presently, we can obtain Exc-B from cultured-type Formosan gorgonian coral *B. excavatum*, which is cultured by the National Museum of Marine Biology and Aquarium (NMBA) in Taiwan. Moreover, in a previous study, we demonstrated the anti-inflammatory and anti-nociceptive effects of Exc-B on intra-plantar carrageenan-induced inflammatory responses [38]. As it is well known that the inflammatory process also plays a critical role in RA progression and in therapeutic strategies, in the present study we sought to examine the anti-rheumatic effects of Exc-B and evaluated the possible mechanisms involved.

## 2. Results

### 2.1. Exc-B Inhibits LPS-Induced Osteoclast-Like Cell Formation

**Figure 1:**
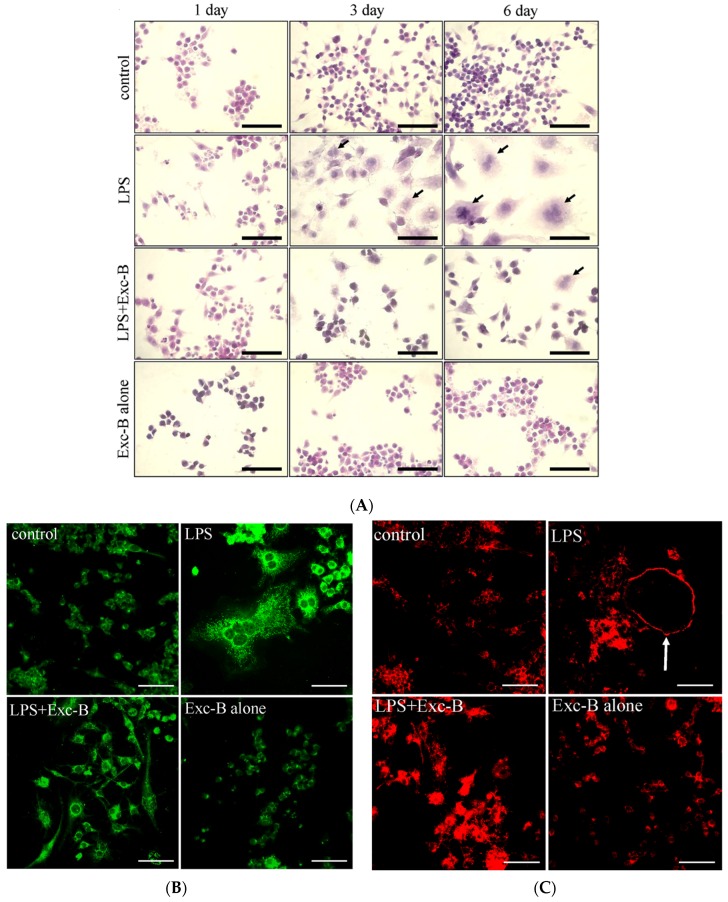

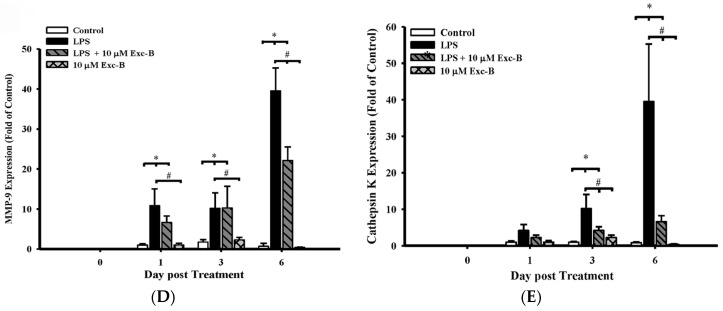
The effects of Exc-B in LPS-stimulated osteoclast-like cells. (**A**) RAW 264.7 cells were cultured for one, three and six days with 10 μM Exc-B in the presence of 10 ng/mL LPS. Haematoxylin and eosin staining was performed after fixation; (**B**) Immunofluorescence microscope photographs of TRAP in LPS-stimulated osteoclast-like cells treated with 10 μM Exc-B for six says and quantification of TRAP immunoreactivity in LPS-stimulated osteoclast-like cells; (**C**) Immunofluorescence microscope photographs of actin rings in LPS-stimulated osteoclast-like cells treated for six says with 10 μM Exc-B and the number of actin rings in LPS-stimulated osteoclast-like cells. Effect of Exc-B on MMP-9 (**D**); and cathepsin K mRNA (**E**) in LPS-stimulated osteoclast-like cells. RAW 264.7 cells were cultured for one, three and six days with 10 μM Exc-B in the presence of 10 ng/mL LPS. The mRNA levels of MMP-9 and cathepsin K were normalized to GAPDH levels. The data are representative of three independent experiments. Each experiment was repeated 4–6 times. Scale bar = 50 μm. Values reflect the mean ± SEM for each group. The data were analysed by one-way analysis of variance (ANOVA) followed by the Student–Newman–Keuls post hoc test. * *p* < 0.05 compared with the control group; # *p* < 0.05 compared with the LPS treatment alone.

In a previous study, we found that Exc-B could inhibit inducible nitric oxide synthase (iNOS) and cyclooxygenase-2 (COX-2) gene and protein expression in LPS-induced RAW 264.7 cells in an in vitro model. Here, we used LPS-induced osteoclast-like cell formation to assess the anti-arthritic activity of Exc-B (10 μM) in vitro. Figure 1A shows the time course of LPS-induced osteoclast-like cell formation at Day 3 and Day 6 compared with the control (arrow). Multinucleated cell formation was visibly reduced when treated with LPS + Exc-B (10 μM). No obvious multinucleated cell formation could be observed from Day 1 to Day 6 when treated with Exc-B alone. Figure 1B,C show the TRAP protein expression and actin ring formation, respectively. Figure 1B indicates that LPS effectively upregulated TRAP protein expression, and Exc-B reduced TRAP protein expression in LPS-induced osteoclast-like cells at Day 6. Quantitative analysis of TRAP protein expression in LPS-induced osteoclast-like cells also showed that TRAP protein expression was reduced with Exc-B treatment at Day 6 (Figure S1A). Figure 1C indicates that LPS effectively promoted actin ring formation (arrow), and Exc-B reduced actin ring formation at Day 6. Quantitative analysis of the number of actin rings in LPS-induced osteoclast-like cells showed that these were decreased by Exc-B at Day 6 (Figure S1B). There was, however, no significant TRAP expression or actin ring formation with Exc-B treatment alone at Day 6 compared to the control (Figure S1). Furthermore, Figure 1 shows the MMP-9 and cathepsin K mRNA expression as determined using qPCR analysis. Figure 1D shows the changes in MMP-9 elicited by LPS-induced osteoclast-like cells at Days 0, 1, 3, and 6; whereby, MMP-9 mRNA expression was significantly increased at Days 1, 3, and 6 when compared to the control, and Exc-B significantly downregulated MMP-9 mRNA expression at Day 6. Similarly, cathepsin K mRNA expression significantly increased at Days 3 and 6 compared to the control, and Exc-B significantly reduced cathepsin K mRNA expression at Days 3 and 6 (Figure 1E). There was, however, no significant change in MMP-9 and cathepsin K mRNA expression with Exc-B (10 μM) treatment alone when compared to the control.

### 2.2. Effect of Exc-B on the Clinical Features of RA

**Figure 2:**
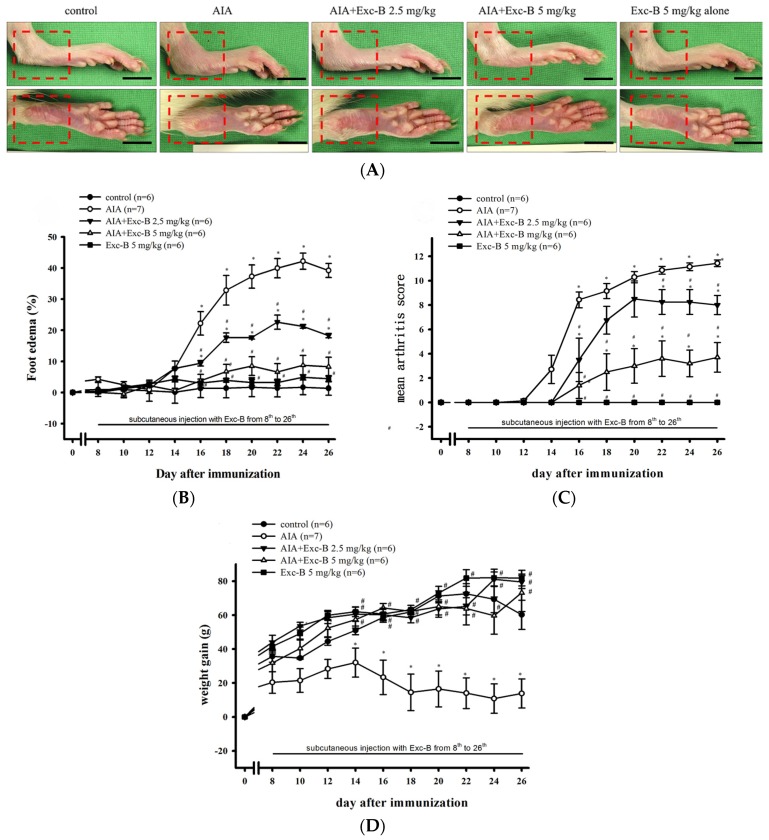
Effect of Exc-B in AIA rats. (**A**) Representative macroscopic photographs of the ankles and paws of the control, AIA, AIA + Exc-B (2.5 mg/kg), AIA + Exc-B (5 mg/kg), and Exc-B alone groups. The AIA and AIA + Exc-B (2.5 mg/kg) groups display significant oedema on the ankle joints and erythema on the hindpaws (red square) in comparison to the control group. The AIA + Exc-B (5 mg/kg) group demonstrate an apparent reduction in AIA-related oedema and erythema. Quantitative analysis of the effect Exc-B at doses of 2.5 or 5 mg/kg on: AIA-related paw oedema (**B**); arthritis scores (**C**); and body weight (**D**). Values reflect the mean ± SEM for each group. Scale bar = 1 cm. The data were analysed by Kruskal–Wallis One Way Analysis of Variance analysis. * *p* < 0.05, significantly different from the control group; # *p* < 0.05, significantly different from the AIA group.

To demonstrate the anti-arthritic activity of Exc-B in vivo, we developed AIA and CIA models with Lewis rats. Rats in the Exc-B treatment groups received subcutaneous injections of Exc-B (2.5 or 5 mg/kg for AIA rats and 5 mg/kg for CIA rats) one time at two-day intervals between Days 8 and 26 after immunization. The clinical features of RA developed rapidly in the AIA rats. Figure 2A depicts the typical representative areas in macroscopic photographs. Both AIA and AIA + Exc-B (2.5 mg/kg) had oedema and erythema of the ankle joints and hindpaws (Figure 2A; red squre) at Day 27 after immunization, while the AIA + Exc-B (5 mg/kg) group had significantly attenuated oedema and erythema of the hindpaws after immunization (Figure 2A). Figure 2B illustrates the time-dependent increase of paw oedema in the AIA groups and foot oedema significantly increased, relative to baseline values, from Days 16 to 26 in the untreated AIA group. The AIA + Exc-B (2.5 or 5 mg/kg) groups demonstrated dose-dependent inhibition of paw oedema from Days 16 to 26 when compared with the untreated AIA group. Quantitative analysis using the macroscopic scoring system revealed a dose-dependent therapeutic effect of Exc-B (2.5 or 5 mg/kg) with regards to the AIA-related increase in arthritis score (Figure 2C). Figure 2D demonstrates that weight gain significantly decreased from Days 16 to 26 in the untreated AIA group, while Exc-B treatment (2.5 or 5 mg/kg) significantly improved weight gain in the treated AIA groups.

**Figure 3:**
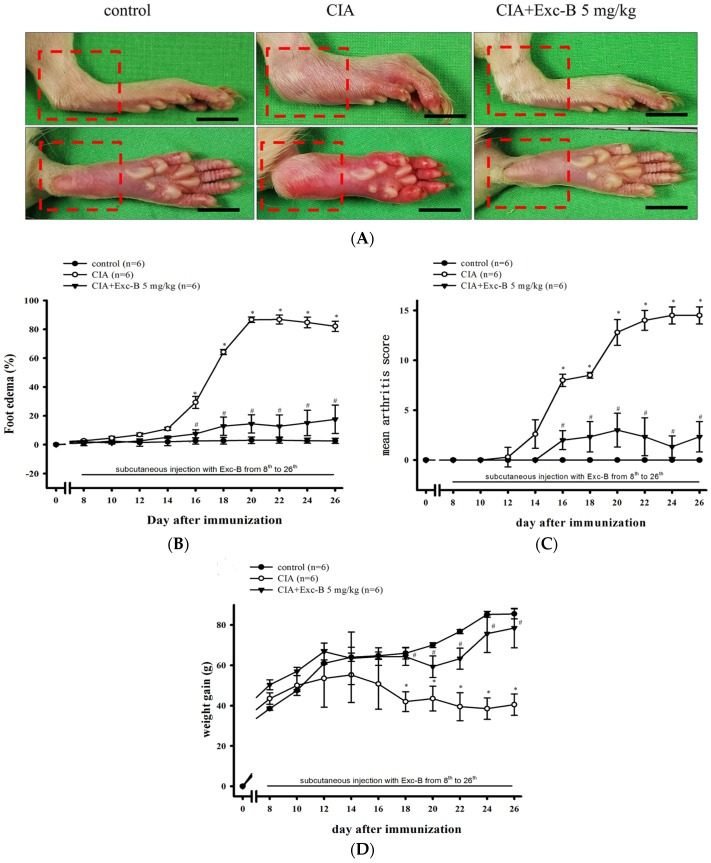
Effect of Exc-B in CIA rats. (**A**) Representative macroscopic photographs of the ankles and paws of the control, CIA, and CIA + Exc-B (5 mg/kg) groups. The CIA group displays significant oedema on the ankle joints and erythema on the hindpaws (red square) in comparison to the control group. The CIA + Exc-B (5 mg/kg) group demonstrate an apparent reduction in CIA-related oedema and erythema. Quantitative analysis of the effect Exc-B at doses of 5 mg/kg on: CIA-related paw oedema (**B**); arthritis scores (**C**); and body weight (**D**). Values reflect the mean ± SEM for each group. Scale bar = 1 cm. The data were analysed by Kruskal–Wallis One Way Analysis of Variance analysis. * *p* < 0.05, significantly different from the control group; # *p* < 0.05, significantly different from the CIA group.

Figure 3 shows a similar effect of Exc-B in CIA rats. Figure 3A shows oedema and erythema of the ankle joints and hindpaws in the CIA group (red square) in comparison to the control group, while the CIA + Exc-B (5 mg/kg) group had significantly attenuated oedema and erythema after immunization at Day 27. Figure 3B illustrates the time-dependent increase in oedema in the CIA group whereby foot oedema significantly increased, relative to baseline values, from Days 16 to 26 in the untreated CIA group. The CIA + Exc-B (5 mg/kg) group demonstrated dose-dependent inhibition in foot oedema compared with the CIA group. The macroscopic scoring system also revealed a therapeutic effect of Exc-B (5 mg/kg) on CIA-related increases in arthritis score (Figure 3C). Figure 3D demonstrates a significant decrease in weight gain from Days 18 to 26 in the untreated CIA group, while in the CIA + Exc-B (5 mg/kg) group the treatment significantly improved weight gain, in comparison to the untreated CIA group. In subsequent evaluations, we focused on histopathologic analysis of the ankle joints.

### 2.3. Effect of Exc-B on Histological Features of RA

**Figure 4:**
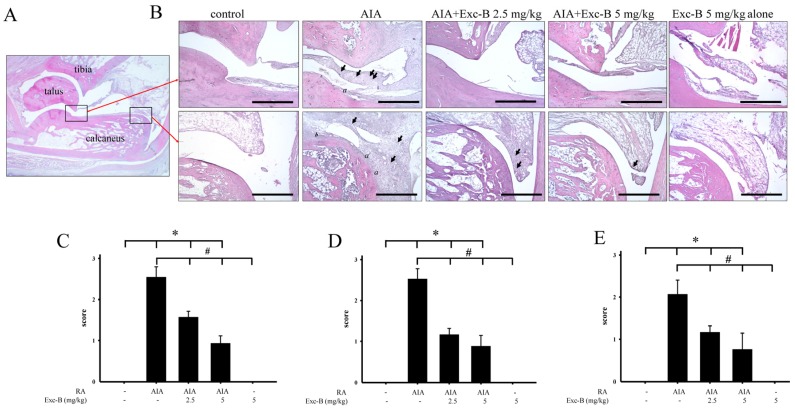
Histopathological assessments of the effect of Exc-B on the ankle joints in AIA rats. (**A**) Normal joint structure showing calcaneus-talus articulation with the distal tibia and normal synovial tissue in the control group; (**B**) Representative haematoxylin and eosin staining images of the ankle joint sections from the control, AIA, AIA + Exc-B (2.5 mg/kg), AIA + Exc-B (5 mg/kg), and Exc-B alone groups of similar areas as outlined by the boxes in the image of the normal joint structure. The AIA group exhibits synovial inflammation (arrows), bone erosion (*b*), and cartilage destruction (*a*). The representative histopathological scores for: synovial inflammation (**C**); cartilage destruction (**D**); and bone erosion (**E**) were assessed in ankle joints of the control, AIA, AIA + Exc-B (2.5 mg/kg), AIA + Exc-B (5 mg/kg), and Exc-B groups. Scale bar = 200 μm. Values reflect the mean ± SEM for each group. *n* = 6 rat per group. The data were analysed by one-way analysis of variance (ANOVA) followed by the Student–Newman–Keuls post hoc test. * *p* < 0.05, significantly different from the control group; # *p* < 0.05, significantly different from the AIA group.

For assessments of the ankle joints, histopathological changes, including synovial inflammation, cartilage destruction, and bone erosion, were scored. Representative photographs of the ankle joint sections from the control, AIA, AIA + Exc-B (2.5 mg/kg), AIA + Exc-B (5 mg/kg), and Exc-B alone (5 mg/kg) groups are shown in Figure 4A, respectively. Normal joint structures exhibiting calcaneus-talus articulation with the distal tibia and normal synovial tissue were observed in the control group (Figure 4A). Rats with AIA displayed marked synovial inflammation (arrow), cartilage destruction (*a*), and bone erosion (*b*) on Day 27 (Figure 4B). In the AIA + Exc-B (2.5 or 5 mg/kg) group, a lower degree of synovial inflammation, cartilage destruction, and bone erosion was observed (Figure 4B–E). No significant changes were observed in the group treated with Exc-B alone.

**Figure 5:**
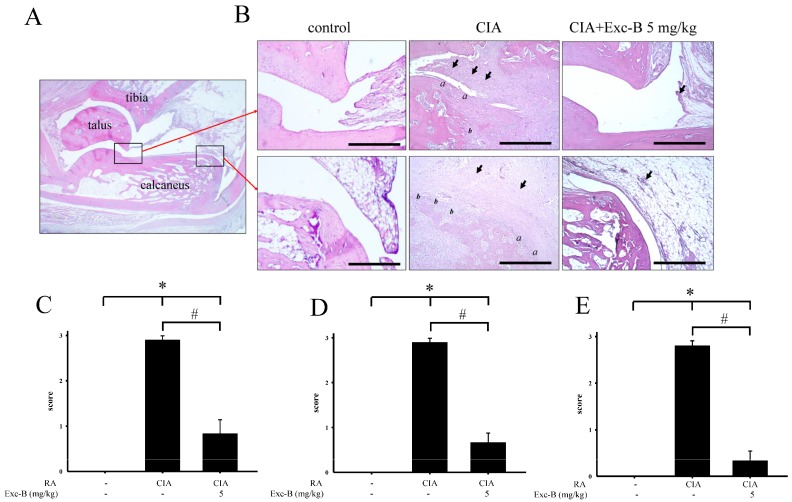
Histopathological assessments of the effect of Exc-B on the ankle joints in CIA rats. (**A**) Normal joint structure showing calcaneus-talus articulation with the distal tibia and normal synovial tissue in the control group; (**B**) Representative haematoxylin and eosin staining images of the ankle joint sections from the control, CIA, and CIA + Exc-B (5 mg/kg) groups in similar areas as outlined by the boxes in the image of the normal joint structure. The CIA group exhibits synovial inflammation (arrows), cartilage destruction (*a*) and bone erosion (*b*). The representative histopathological scores for: synovial inflammation (**C**); cartilage destruction (**D**); and bone erosion (**E**) were assessed in ankle joints of the control, CIA, and CIA + Exc-B (5 mg/kg) groups. Scale bar = 200 μm. Values reflect the mean ± SEM for each group. *n* = 6 rat per group. The data were analysed by one-way analysis of variance (ANOVA) followed by the Student–Newman–Keuls post hoc test. * *p* < 0.05, significantly different from the control group. # *p* < 0.05, significantly different from the CIA group.

In CIA rats, similar results were observed, whereby untreated CIA rats exhibited marked synovial inflammation (arrow), cartilage destruction (*a*), and bone erosion (*b*) on Day 27 (Figure 5B), while the CIA + Exc-B (5 mg/kg) group demonstrated a significantly lower degree of synovial inflammation, cartilage destruction, and bone erosion when compared with CIA group (Figure 5B–E). Thus, we proceeded with immunohistochemical staining of the ankle tissues and Western blot analysis of the synovial tissue to elucidate the possible mechanism of the effect of Exc-B in RA.

### 2.4. Effect of Exc-B on TRAP-Positive Multinucleated Cell Formation In Vivo

**Figure 6:**
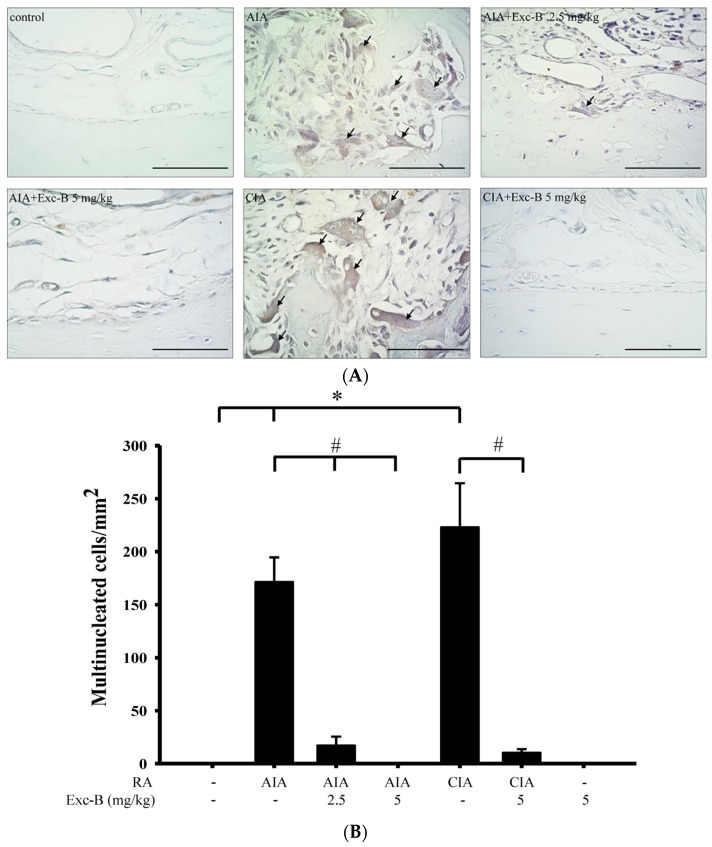
Effect of Exc-B on TRAP-positive multinucleated cell formation in vivo. Representative high-power field (400×) magnifications of specimens (**A**) from the: control; AIA; CIA; AIA + Exc-B (2.5 mg/kg); AIA + Exc-B (5 mg/kg); and CIA+ Exc-B (5 mg/kg) groups. The CIA and AIA groups show the TRAP-positive multinucleated cells (arrows) in the ankle joints sections. Quantitative analysis of the number of multinucleated cells (**B**). Values reflect the mean ± SEM for each group. Scale bar = 50 μm. *n* = 6 rat per group. The data were analysed by one-way analysis of variance (ANOVA) followed by the Student–Newman–Keuls post hoc test. * *p* < 0.05, significantly different from the control group; # *p* < 0.05, significantly different from the AIA or CIA group.

To investigate the effect of Exc-B on the number of TRAP-positive multinucleated cells, ankle joint sections were stained for the TRAP protein with haematoxylin (Figure 6). Figure 6A shows the distribution of TRAP-positive multinucleated cells on the bone surface of the ankle joint from the control, AIA, CIA, AIA + Exc-B (2.5 mg/kg), AIA + Exc-B (5 mg/kg), and CIA + Exc-B (5 mg/kg) groups. TRAP-positive multinucleated cells appeared to increase in the ankle joints of the AIA, AIA + Exc-B (2.5 mg/kg), and CIA groups. In the AIA + Exc-B (5 mg/kg) and CIA + Exc-B (5 mg/kg) groups, the treatment with Exc-B effectively inhibited TRAP-positive multinucleated cell formation (arrow) on the bone surfaces of the ankle joints. Quantitative analysis of TRAP-positive multinucleated cell formation also showed that the number of TRAP-positive multinucleated cells in the AIA and CIA groups was significantly increased (Figure 6B). However, Exc-B markedly inhibited both AIA- and CIA-related increases in TRAP-positive multinucleated cells in the ankle joints of the arthritic rats. No significant TRAP-positive multinucleated cell formation was found in the Exc-B (5 mg/kg) treatment alone group when compared with the control group (Figure 6A). Thus, treatment with Exc-B showed a significant reduction in the number of osteoclast-like cells.

### 2.5. Effect of Exc-B on NFATc1 in Arthritic Rats

**Figure 7:**
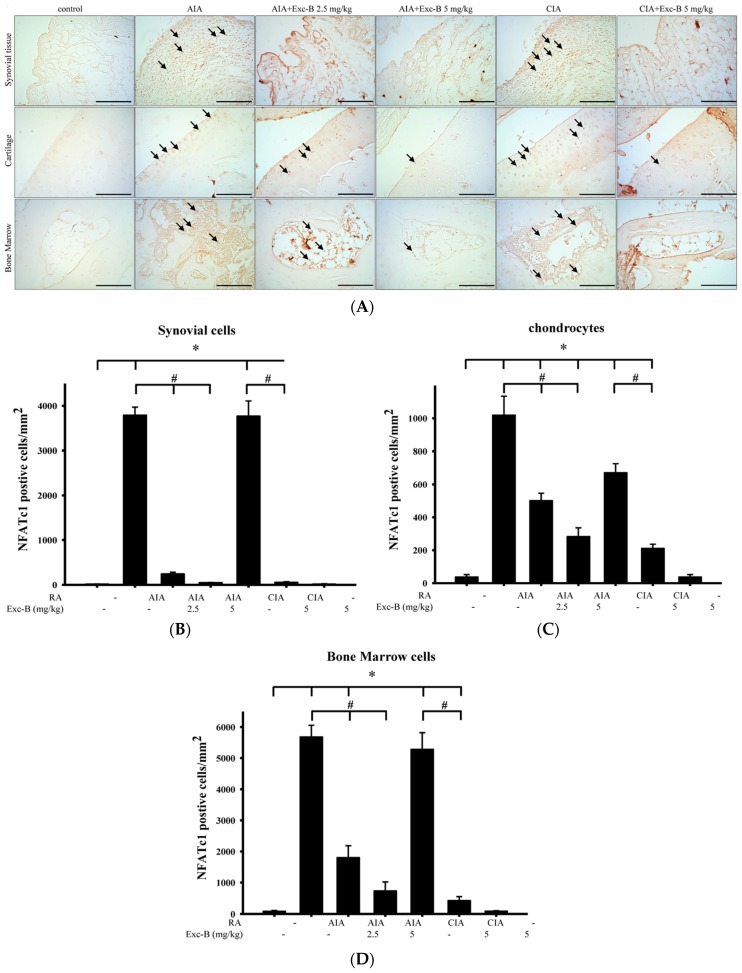
Effect of Exc-B on NFATc1 protein expression. (**A**) NFATc1 protein immunoreactivity is indicated in red-brown (arrows) in ankle joint sections from the control, AIA, CIA, AIA + Exc-B (2.5 mg/kg), AIA + Exc-B (5 mg/kg) and CIA + Exc-B (5mg/kg) groups. NFATc1 immunoreactivity in the articular cartilage, synovial tissue, and bone marrow, respectively, and the quantification of NFATc1 positive cells in the: articular cartilage (**B**); synovial tissue (**C**); and bone marrow (**D**) are shown. SB, subchondral bone; BM, bone marrow. Values reflect the mean ± SEM for each group. Scale bar = 100 μm. *n* = 6 rat per group. The data were analysed by one-way analysis of variance (ANOVA) followed by the Student–Newman–Keuls post hoc test. * *p* < 0.05, significantly different from the control group; # *p* < 0.05, significantly different from the AIA or CIA group.

To investigate the effect of Exc-B on the number of NFATc1 positive cells, ankle joint sections were stained for NFATc1—a key transcription factor for osteoclast differentiation. Figure 7 shows the distribution of NFATc1 immunoreactivity in the ankle joint sections from the control, AIA, CIA, AIA + Exc-B (2.5 mg/kg), AIA + Exc-B (5 mg/kg), CIA + Exc-B (5 mg/kg), and Exc-B (5 mg/kg) alone groups. NFATc1 immunoreactivity appeared to increase in the synovial tissue, cartilage, and bone marrow of the ankle joints in the AIA and CIA groups (arrow). In the AIA + Exc-B (2.5 mg/kg), AIA + Exc-B (5 mg/kg), and CIA + Exc-B (2.5 mg/kg) groups, the treatment significantly reduced NFATc1 immunoreactivity in the articular cartilage, synovial tissue, and bone marrow of the ankle joints. Quantitative analysis of NFATc1 positive cells in synovial tissue, articular cartilage, and bone marrow also show marked upregulation of NFATc1 immunoreactivity in the AIA and CIA groups (Figure 7B–D). However, Exc-B markedly inhibited the induced upregulation of NFATc1 positive cell expression in synovial tissue, articular cartilage, and bone marrow in the treated AIA and CIA groups. There was, however, no difference between the group treated with Exc-B (5 mg/kg) alone and the control group (Figure 7B–D).

### 2.6. Effect of Exc-B on Cathepsin K, MMP-2, MMP-9, and CD11b in Synovial Tissue

**Figure 8:**
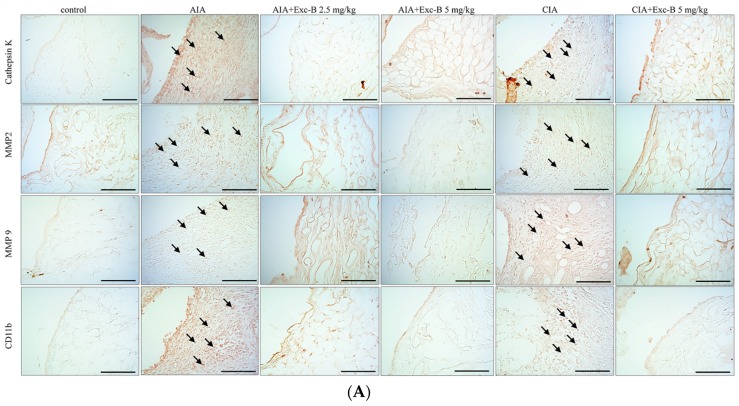

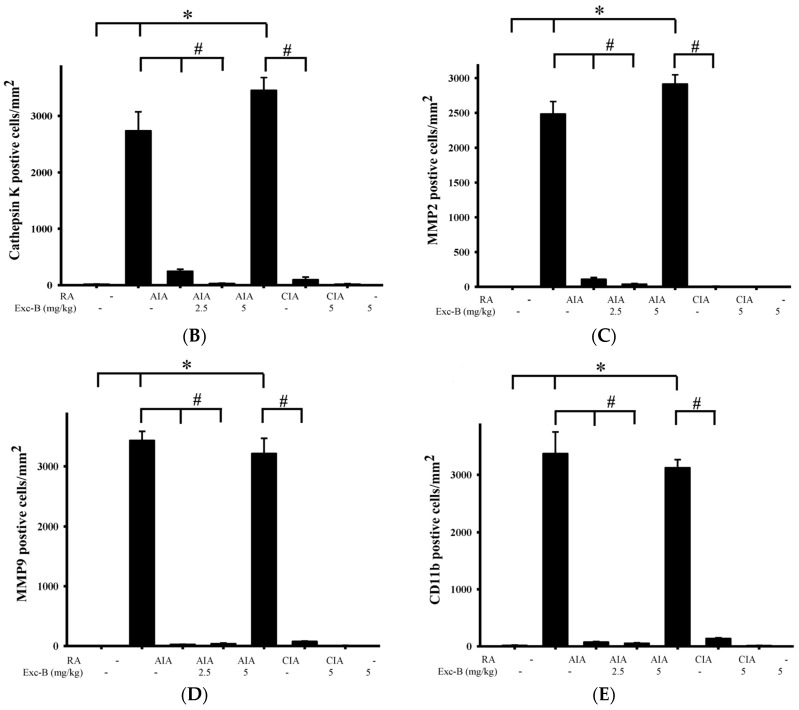
Effect of Exc-B on cathepsin K, MMP-2, MMP-9, and CD11b protein expression in synovial tissue. (**A**) Immunoreactivity is indicated in red-brown (arrows) in ankle joint sections from the control, AIA, CIA, AIA + Exc-B (2.5 mg/kg), AIA + Exc-B (5 mg/kg) and CIA + Exc-B (5 mg/kg) groups. Quantitative analysis of: cathepsin K (**B**); MMP-2 (**C**); MMP-9 (**D**); and CD11b (**E**) positive cells in synovial cells is shown. Values reflec the mean ± SEM for each group. Scale bar = 100 μm. *n* = 6 rat per group. The data were analysed by one-way analysis of variance (ANOVA) followed by the Student–Newman–Keuls post hoc test. * *p* < 0.05, significantly different from the control group; # *p* < 0.05, significantly different from the AIA or CIA group.

Figure 8 shows the distribution of cathepsin K, MMP-2, MMP-9, and CD11b in synovial tissues from the control, AIA, CIA, AIA + Exc-B (2.5 mg/kg), AIA + Exc-B (5 mg/kg), CIA + Exc-B (5 mg/kg), and Exc-B (5 mg/kg) alone groups. Cathepsin K appeared to increase in synovial tissues of the ankle joints in the AIA and CIA groups, while the AIA + Exc-B (2.5 mg/kg), AIA + Exc-B (5 mg/kg), and CIA + Exc-B (2.5 mg/kg) groups show significant inhibition of cathepsin K immunoreactivity in synovial tissues of the ankle joints. Quantitative analysis of the number of cathepsin K positive cells also showed marked upregulation of immunoreactivity (Figure 8B). However, Exc-B inhibited the upregulation of cathepsin K positive cells in the AIA and CIA groups. Similarly, MMP-2 appeared to increase in synovial tissues of the ankle joints in the AIA and CIA groups, and Exc-B significantly inhibited MMP-2 immunoreactivity (Figure 8A). Quantitative analysis of the number of MMP-2 positive cells in the synovial tissue also showed marked upregulation of immunoreactivity in the AIA and CIA groups, and a reduction in the number of MMP-2 positive cells with Exc-B treatment (Figure 8C). The immunoreactivity of MMP-9 could also be observed in the synovial tissues of the ankle joints from the AIA and CIA groups, and Exc-B treatment showed significant inhibition of MMP-9 immunoreactivity in the synovial tissue (Figure 8A). The same finding was also confirmed by quantitative analysis of MMP-9 immunoreactivity (Figure 8D). CD11b immunoreactivity was also evident in the synovial tissues of the AIA and CIA groups, and Exc-B treatment showed strong inhibition of CD11b protein expression in the synovial tissue (Figure 8A). Quantitative analysis of CD11b positive cells showed that Exc-B significantly reduced the number of CD11b positive cells (Figure 8E). There were no differences in the number of cathepsin K, MMP-2, MMP-9, and CD11b positive cells between the groups treated with Exc-B (5 mg/kg) alone and the control group (Figure 8B–E).

### 2.7. Effect of Exc-B on Cathepsin K, MMP-2, and MMP-9 in Cartilage

**Figure 9:**
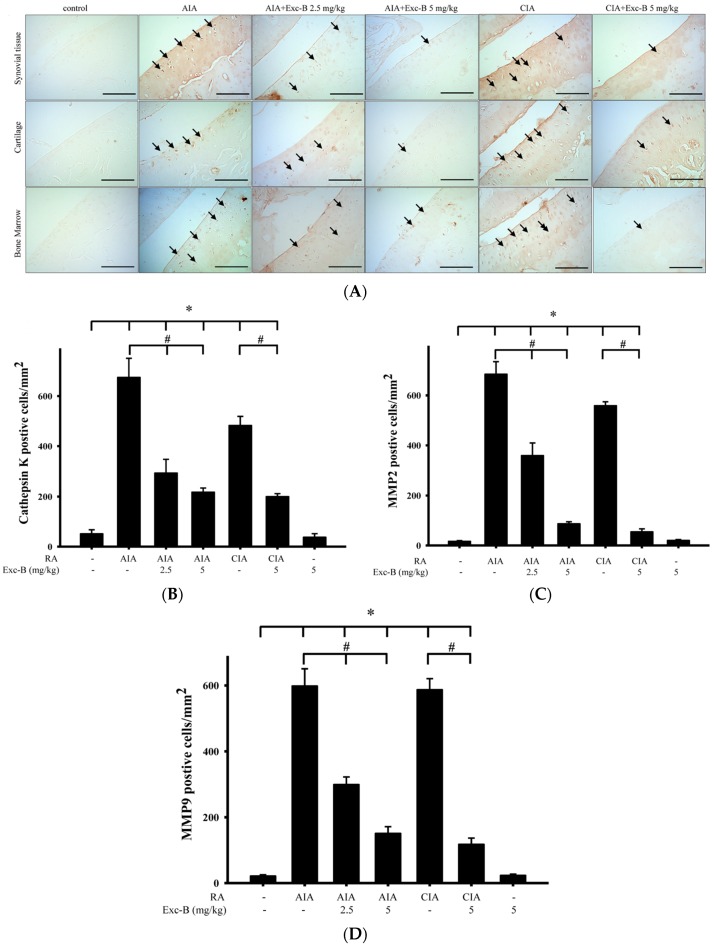
Effect of Exc-B on cathepsin K, MMP-2, MMP-9 protein expression in cartilage. (**A**) Immunoreactivity is indicated in red-brown (arrows) in ankle joint sections from the control, AIA, CIA, AIA + Exc-B (2.5 mg/kg), AIA + Exc-B (5 mg/kg) and CIA + Exc-B (5 mg/kg) groups. Quantitative analysis of: cathepsin K (**B**); MMP-2 (**C**); and MMP-9 (**D**) positive cells in chondrocytes is shown. Values reflect the mean ± SEM for each group. Scale bar = 100 μm. *n* = 6 rat per group. The data were analysed by one-way analysis of variance (ANOVA) followed by the Student–Newman–Keuls post hoc test. * *p* < 0.05, significantly different from the control group. # *p* < 0.05, significantly different from the AIA or CIA group.

In RA, cytokine, protease, and matrix metalloproteinase expressions influence chondrocyte metabolism, which can lead to cartilage damage and bone erosion. Thus, we sought to investigate the effect of Exc-B in the cartilage of the ankle joints. Figure 9 shows the distribution of cathepsin K, MMP-2, and MMP-9 in cartilage from the control, AIA, CIA, AIA + Exc-B (2.5 mg/kg), AIA + Exc-B (5 mg/kg), CIA + Exc-B (5 mg/kg), and Exc-B (5 mg/kg) alone groups. Cathepsin K appeared to increase in the cartilage of the ankle joints from the AIA and CIA groups, while the AIA + Exc-B (2.5 mg/kg), AIA + Exc-B (5 mg/kg), and CIA + Exc-B (2.5 mg/kg) groups showed significant inhibition of cathepsin K immunoreactivity in the cartilage. Quantitative analysis of the number of cathepsin K positive cells in cartilage also showed marked upregulation of immunoreactivity (Figure 9B) that was inhibited with Exc-B treatment. MMP-2 appeared to be increased in cartilage of AIA and CIA groups, and Exc-B treatment significantly inhibited MMP-2 immunoreactivity (Figure 9A). Quantitative analysis of the number of MMP-2 positive cells in cartilage also shows an AIA and CIA-related upregulation of immunoreactivity, and a marked reduction in the number of MMP-2 positive cells with Exc-B treatment (Figure 9C). Similar findings with regards to the immunoreactivity of MMP-9 were observed in cartilage of the AIA and CIA groups, as well as with Exc-B treatment, which were confirmed by quantitative analysis (Figure 9D). There was no difference in the number of cathepsin K, MMP-2, and MMP-9 positive cells between the group treated with Exc-B (5 mg/kg) alone group and the control group (Figure 9B–D).

### 2.8. Effect of Exc-B on MAPK, HO-1, and HMGB-1 Protein Expression in Synovial Tissue

**Figure 10:**
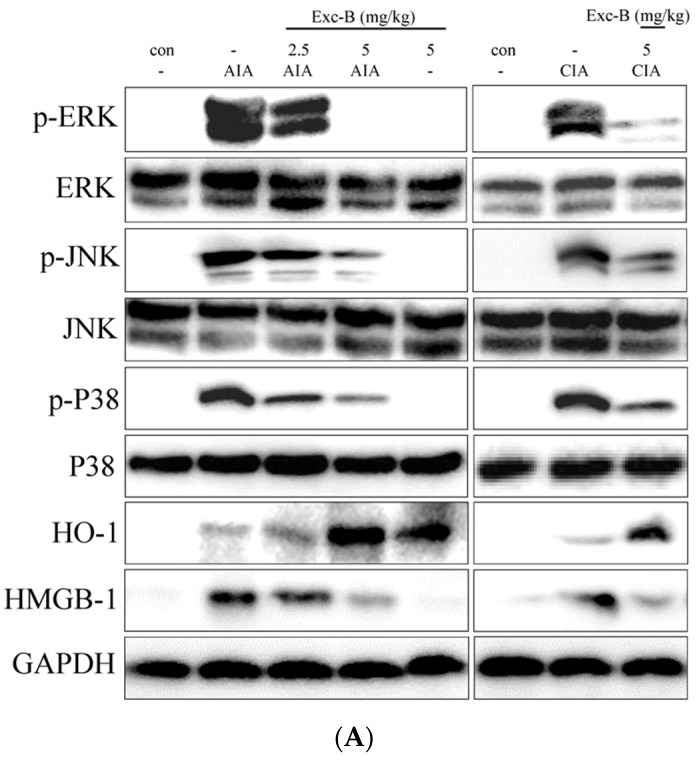

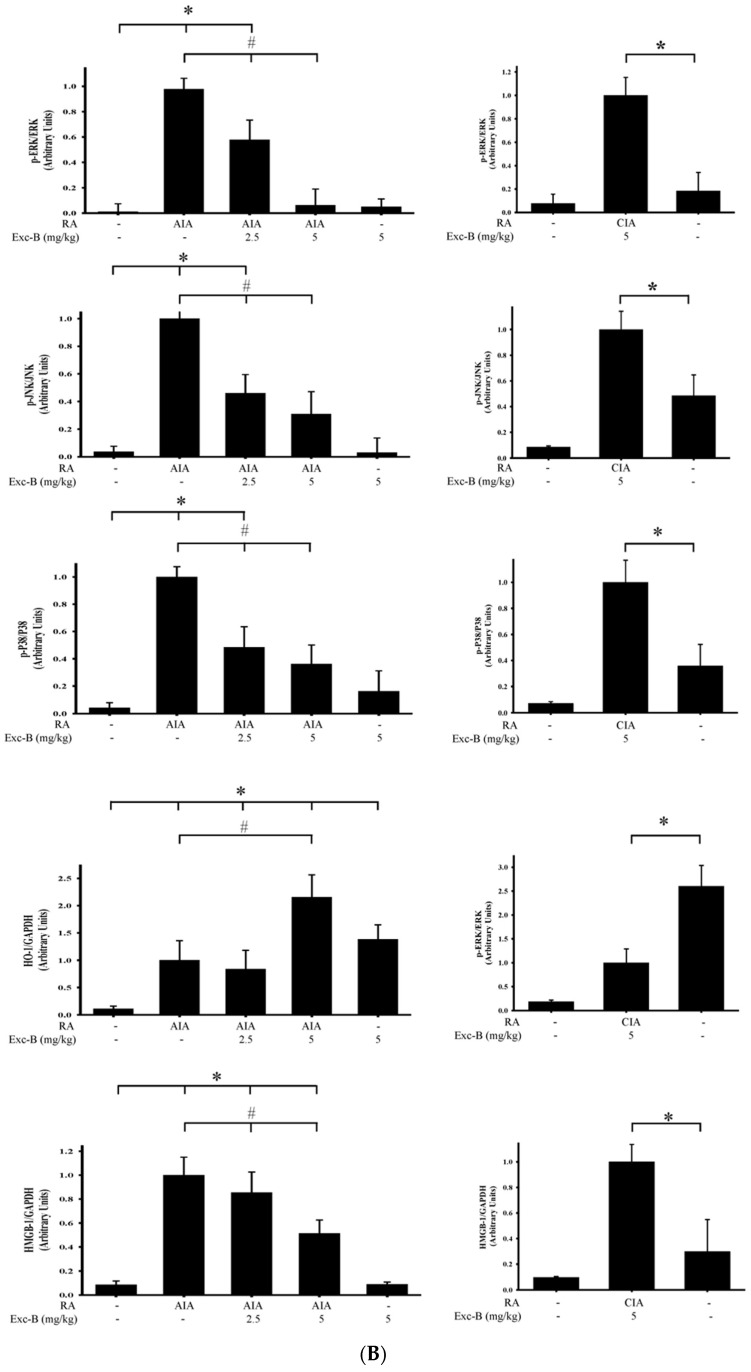
Effect of Exc-B on MAPK, HO-1, and HMGB-1 in homogenized knee synovial tissues. Western blot analysis of the effects of subcutaneous Exc-B administration on protein expression of HO-1, HMGB-1, and MAPK signalling, as well as GAPDH in knee synovial tissue homogenates from rats with: AIA (**A**); or CIA (**B**). Exc-B (2.5 or 5 mg/kg) significantly inhibits HMGB-1 and MAPK signalling and upregulates HO-1 protein expression after immunization. The There is no difference between groups in the protein expression of GAPDH in synovial tissues. Quantification values reflect the mean ± S.E.M. of three different experiments. *n* = 6 rat per group. The data were analysed by one-way analysis of variance (ANOVA) followed by the Student–Newman–Keuls post hoc test. * *p* < 0.05, significantly different from the control group. # *p* < 0.05, significantly different from the AIA or CIA group.

Western blot analysis was used to assess the effect of Exc-B and its possible mechanism of action in RA. Knee synovial tissues were collected from the control, AIA, CIA, AIA + Exc-B (2.5 mg/kg), AIA + Exc-B (5 mg/kg), CIA + Exc-B (5 mg/kg), and Exc-B (5 mg/kg) alone groups at Day 27 after immunization. The protein expression of p-ERK, ERK, p-P38, P38, p-JNK, JNK, HO-1, and HMGB-1 is displayed in Figure 10. The protein expression and quantitative result reveal that p-ERK, p-P38, p-JNK, HO-1, and HMGB-1 was upregulated in synovial tissues after Day 27 of immunization in comparison to the control group (Figure 10A). Subcutaneous injections of Exc-B (2.5 or 5 mg/kg) significant reduced p-ERK, p-P38, p-JNK, HMGB-1 protein expression, and upregulated HO-1 protein expression in synovial tissues of the treated AIA rats. Similar findings were also confirmed by Western blot analysis of tissues from the CIA + Exc-B (5 mg/kg) group (Figure 10A). There were, however, no observed changes in total ERK, JNK, or P38 in the synovial tissues. Administration of Exc-B 5 mg/kg alone also had no observable effects on the upregulation of p-ERK, p-P38, p-JNK, HMGB-1 protein expression, with the exception of HO-1 protein expression (Figure 10). Hence, Exc-B had a significant effect on the MAPK and HO-1/HMGB-1 pathways in synovial tissues after immunization.

### 2.9. Effect of Exc-B on M-CSF and IL-17A of Blood Serum

**Figure 11:**
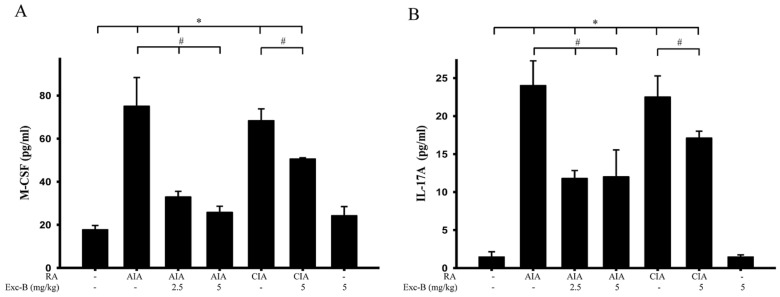
Effect of Exc-B on the levels of M-CSF and IL-17A in blood serum: (**A**) effect of Exc-B on the level of M-CSF in the blood serum of control, AIA, CIA, and Exc-B treated rats; and (**B**) effect of Exc-B on the level of IL-17A in the blood serum of control, AIA, CIA, and Exc-B treated rats. Values reflect the mean ± SEM for each group. *n* = 6 rat per group. The data were analysed by one-way analysis of variance (ANOVA) followed by the Student–Newman–Keuls post hoc test. * *p* < 0.05, significantly different from the control group; # *p* < 0.05, significantly different from the AIA or CIA group.

It is known that increases in M-CSF and IL-17A could lead to osteoclastogenesis. We, therefore, sought to measure the serum levels of M-CSF and IL-17A in the control, AIA, CIA, AIA + Exc-B (2.5 mg/kg), AIA + Exc-B (5 mg/kg), CIA + Exc-B (5 mg/kg), and Exc-B (5 mg/kg) alone groups at Day 27 after immunization. The level of these pro-inflammatory cytokines were analysed using a multiplex immunoassay. Consistent with the progression of RA and upregulation of TRAP-positive multinucleated cells, the levels of M-CSF and IL-17A in the AIA and CIA groups were significantly increased in the blood serum samples. However, treatment with 2.5 or 5 mg/kg Exc-B showed a marked decrease in the levels of M-CSF and IL-17A at Day 27 in AIA rats after immunization (Figure 11). Analogous results were observed in the treated CIA rats, in that the level of M-CSF and IL-17A decreased with 5 mg/kg Exc-B treatment (Figure 11). Thus, Exc-B could reduce osteoclastogenesis in RA via the downregulation of the pro-inflammatory cytokines M-CSF and IL-17A.

## 3. Discussion

### 3.1. Summary

The purpose of the present study was to analyse the anti-osteoclastic activity and anti-arthritic effect of Exc-B in in vitro and in vivo arthritic models, respectively. In a previous study, we showed that Exc-B significantly downregulated the protein expression of iNOS and COX-2 in LPS-stimulated RAW 264.7 murine macrophage cells. In this study, we found that Exc-B also significantly inhibited multinucleated cell formation with the reduction of MMP-9 and cathepsin K mRNA expression, and TRAP expression and actin ring formation in LPS-induced osteoclast-like cells. Moreover, administration of Exc-B significantly reduced AIA and CIA-related ankle oedema, arthritis scores, and weight loss in in vivo models. Histopathological and immunohistochemical examination further illustrated that synovial inflammation, cartilage destruction, and bone erosion, with the upregulation of osteoclast-related protein expressions of cathepsin K, MMP-2, MMP-9, NFATc1, and CD11b in ankle joint tissues of the AIA and CIA rats, was also significantly reduced with Exc-B treatment. Moreover, Exc-B significantly reduced the number of TRAP-positive multinucleated cells, which could have affected the MAPK pathway and HMGB-1 protein expression in the synovial tissues and reduced the level of M-CSF and IL-17A in blood serum. Thus, systemic injection of Exc-B not only attenuated the pathological changes of RA in the ankle joint, but also significantly inhibited osteoclast-related protein expressions.

### 3.2. Anti-Inflammatory Effect of Exc-B In Vitro and In Vivo

The ant-inflammatory and immunodulatory actions of Exc-B have been observed in TPA-induced inflammation and dermatitis in murine skin, as well as in LPS-induced mouse bone marrow-derived dendritic cells, with the reduction of IL-6 and TNF-α protein expressions [37]. In a previous study, we also examined the anti-inflammatory potential of Exc-B in LPS-induced RAW 264.7 cells with downregulated iNOS and COX-2 gene and protein expression [38]. It is well known that chronic inflammation plays an important role in RA, with joint swelling and immune cell infiltration in the synovial tissue. These infiltrating cells, including neutrophils, macrophages, T cells, B cells, dendritic cells, and osteoclasts lead to synovial hyperplasia and pannus formation causing erythema and swelling of the joints [7,39]. Wei et al. indicated that Exc-B could reduce TPA-induced skin oedema by reducing iNOS, COX-2, p-ERK protein expression. Our previous study also revealed that Exc-B could reduce carrageenan-induced paw oedema by downregulating iNOS protein expression and decreasing the number infiltrating cell clusters in paw tissues [38]. Previous studies clearly indicated that AIA could be used as a standard experimental arthritis model for preclinical testing of numerous anti-arthritic agents [40,41]. AIA is also a reliable model for the onset and progression of polyarthritis in which polyarticular inflammation with marked bone erosion are easily measurable [40]. In this study, we also examined the possible therapeutic effects of Exc-B on human rheumatoid arthritis and effects of Exc-B on collagen-induced arthritis (CIA). A previous study indicated that CIA showed more serious and obvious cartilage destruction associated with a stronger immune response on the articular surface, bone resorption, synovial fibroblast proliferation, and more serious inflammatory cell infiltration and periarticular inflammation [40,42]. Previous studies also indicated that CIA lesions are more analogous to human RA than AIA lesions. Our data also showed that foot oedema in the CIA group showed greater swelling and a higher mean arthritis score than in the AIA group. Moreover, the CIA also showed higher pathological scores for synovial inflammation, cartilage destruction, and bone destruction compared to the AIA group (Figure 4 and Figure 5). The present results also show that Exc-B significantly inhibited foot oedema in adjuvant (AIA) and type II collagen-induced arthritic (CIA) rats (Figure 2 and Figure 3). Histological evaluations also demonstrated that Exc-B significantly reduced the level of synovial inflammation in AIA and CIA rats (Figure 4 and Figure 5). Exc-B also reduced MAPK protein expression in knee synovial tissues, which plays a key role in the production of pro-inflammatory cytokines and downstream signalling events leading to inflammation in arthritis [43,44]. Administration of Exc-B had similar therapeutic effects in the two models. Thus, we propose that Exc-B could reduce chronic inflammation in the progression of RA and may be useful for treating human rheumatoid arthritis.

### 3.3. Anti-Osteoclast Activity of Exc-B

Osteoclasts are multinucleated cells, which are mainly differentiated from bone marrow hematopoietic monocyte/macrophage lineages [7,11]. Although, LPS-challenged murine macrophages are widely used for in vitro anti-inflammatory screening of secondary metabolites from terrestrial and marine organisms, LPS could also induce osteoclastogenesis with TRAP-positive multinucleated cell formation from mouse bone marrow and RAW 246.7 murine macrophages [24,45,46,47,48,49,50]. In this study, we used LPS-induced RAW 264.7 cells to form multinucleated cells with upregulated cathepsin K and MMP-9 mRNA expression. Our immunocytochemistry staining also showed upregulated TRAP protein expression and actin ring formation. Furthermore, Exc-B down regulated TRAP protein expression and the number of actin rings formed, and decreased cathepsin K and MMP-9 mRNA expression. The in vivo studies showed that TRAP-positive multinucleated cells played an important role in cartilage damage and bone resorption at the synovial-bone junction in the AIA and CIA rats [51,52]. Histologic analysis revealed bone erosion and cartilage destruction in the ankles of AIA and CIA rats; although, the average scores of bone erosion and cartilage destruction were higher in the CIA rats than in the AIA rats. Exc-B significantly decreased the bone erosion and cartilage destruction scores in both AIA and CIA rats (Figure 5C,D, and Figure 7C,D). Moreover, the upregulation of TRAP-positive multinucleated cells could also be inhibited with Exc-B treatment (Figure 8). Cathepsin K and MMP-9 both play important roles in osteoclastogenesis and osteoclastic activity to cause joint destruction. The levels of cathepsin K and MMP-9 positive cells were both decreased in synovial tissues of the ankle joints with Exc-B (2.5 or 5 mg/kg) treatment. Thus, Exc-B could prevent joint destruction in RA via the inhibition of osteoclast formation and activity.

### 3.4. The Possible Therapeutic Mechanism of Exc-B in RA

NFATc1 is a key transcription factor in osteoclastogenesis [20,21,53]. NFATc1 could via the HO-1/HMGB-1 and MAPK pathway, and auto-amplification to mediate cell fusion and matrix metalloproteinase and cathepsin expressions, lead to osteoclast differentiation and bone resorption in RA [20,54]. Based on immunohistochemistry, in the present study, NFATc1 protein expressions and the number of positive cells in the ankle joints were decreased with Exc-B treatment (Figure 8). Thus we propose that Exc-B could have inhibited cathepsin K, MMP-2, and MMP-9 by diminishing NFATc1 protein expression in the AIA and CIA rats. Moreover, past studies indicate that osteoclastogenesis would result via the HO-1/HMGB-1 and MAPK pathway to mediate NFATc1 protein expression in osteoclast differentiation [20,54]. In this study, we also examined the MAPK protein pathway, and upregulation of MAPK protein expression was detected in knee synovial tissues from the AIA and CIA rats. Exc-B treatment, however, resulted in the downregulation of p-38, p-ERK, and p-JNK in the synovial tissues of the AIA rats, and a similar effect of Exc-B was observed in the CIA rats. Moreover, we also evaluated the HO-1/HMGB-1 pathway. HO-1 is an anti-inflammatory and anti-oxidant agent that inhibits HMGB-1, which has been identified as a cytokine released by osteoclast precursors to promote osteoclastogenesis [54]. Western blot analysis showed increased HO-1 protein expression in synovial tissues from untreated AIA and CIA rats and a reduction in HMGB-1 with Exc-B treatment. Previous studies indicate that pro-inflammatory factors, such as TNF-alpha, IL-6, IL-17A, and CD11b could promote bone resorption by upregulating osteoclast-related proteins [55,56]. These factors could also active MAPK-related pathways in RA and aggravate the progression of RA. However, Wei et al. indicate that Exc-B could reduce TNF-alpha, IL-6 protein expression in LPS-induced mouse BMDCs, and down regulate p-ERK protein in TPA-induced mouse skin. In this study, we therefore, examined the CD11b protein expression in synovial tissues from AIA and CIA rats. The quantitative results show that Exc-B treatment significantly decreased the number of CD11b positive cells. Moreover, the serum level of IL-17A and M-CSF was also reduced with Exc-B treatment. Therefore, Exc-B could reduce osteoclastogenesis via the downregulation of MAPK, IL-17A, and M-CSF, and the upregulation of HO-1 in RA. Furthermore, previous studies have demonstrated that Th17 cells release IL-17 to stimulate the ostoeclastogenesis in RA [17]. In the present study, we found that Exc-B significantly reduced the level of IL-17A in the serum of AIA- and CIA-rats. Our histopathologic observations clearly showed that the expression of inflammatory cells in AIA- and CIA-rats was reduced by Exc-B (Figure 2 and Figure 3). Thus, we predicted that Exc-B could modulate T lymphocyte infiltration by reducing IL-17A protein expression.

### 3.5. Coral Aquaculture for Anti-Inflammatory and Anti-Arthritic Agents

Although coral reef ecosystems comprise one of the most potent marine resources for the discovery and development of novel therapeutic agents, there is still a critical issue with regards to the extensive collection of corals, and the disruption of these ecosystems [32]. Coral aquaculture could, however, overcome the aforementioned limitation and may prompt further drug discovery research [32]. In this study, we continued our previous evaluation of Exc-B though coral aquaculture, and indicated that cultured-type yields could be higher than that of wild-type yields. Moreover, it is more important that we could obtain a substantial quantity of Exc-B with a stable supply from a wild-type gorgonian *B. excavatum* by controlling the lighting and nutrients in the environment [32,38]. To our knowledge, this is the first report of a briarane-type diterpene coral derivative compound that could attenuate the development of RA in experimental animal models of RA by inhibiting osteoclast differentiation. Nevertheless, over 3000 new compounds with different biological activities have been isolated from corals in the past two decades. Coral aquaculture could be a particularly useful method for obtaining large quantities of these marine compounds in order to bypass the complex steps of chemical synthesis. Hence, coral aquaculture has potential and maybe essential for marine drug development.

## 4. Materials and Methods

### 4.1. Extraction of Exc-B

In present study, the extraction and structure of Exc-B was as described in our previous study; Exc-B was isolated from cultured-type Formosan gorgonian coral, *B. excavatum* (obtained from National Museum of Marine Biology & Aquarium (NMMBA), Pingtung, Taiwan; Wet weight: 2.8 kg) [36,38]. Briefly, the cultured-type coral was minced and exhaustively extracted with methanol and dichloromethane (1:1; 8 L) and partitioned into H_2_O and ethyl acetate layers. The ethyl acetate layer was separated by normal phase column chromatography (silica gel 60, 230–400 mesh) and eluted with n-hexane, ethyl acetate, and methanol to yield 30 fractions. Fraction 11 was further purified with normal phase silica gel and eluted with n-hexane-ethyl acetate (1:6) to obtain Exc-B. The structure of the compound was confirmed by nuclear magnetic resonance (NMR) spectroscopy at 400 MHz (Varian Mercury Plus 400 FT-NMR, Palo Alto, CA, USA). The purity (>98%) of Exc-B was identified and verified with the ^1^H-NMR and ^13^C-NMR spectrums and compared to the structures of other natural compounds derived from soft coral, as described previously [36].

### 4.2. LPS-Induced Osteoclast-Like Cell Model

The method of generating osteoclast-like cells was based on that reported by Guo et al. and Islam et al. [24,45]. For osteoclast-like cell induction with LPS (10 ng/mL; Sigma-Aldrich, St. Louis, MO, USA), RAW 264.7 murine macrophages were harvested by pipetting, and seeded at 5 × 10^5^ cells per well in a 6-well plate with DMEM (2 mM glutamine, 1 mM pyruvate, 4.5 g/L glucose, 50 mg/mL streptomycin, and 100 U/mL penicillin G) includingheat-inactivated foetal bovine serum (10%, Gibco fetal bovine serum product, Life Technologies Corporation, Grand Island, NY, USA) at 37 °C in a humidified 5% CO_2_: 95% air incubator for 1, 3, and 6 days. To evaluate the effect of Exc-B on LPS-induced osteoclastogenesis, cells were seeded onto slides and treated with 10 μM Exc-B for 1, 3, and 6 days. Haematoxylin and eosin staining was used to evaluate general and pathological changes in morphology visualized by microscopic examination (DM 6000B, Leica Inc., Leitz-Park, Wetzlar, Germany) using a microscope digital image output system (SPOT idea 5.0 Mp Colour Digital Camera, Diagnostic Instruments Inc., Sterling Heights, MI, USA). For in vitro analysis, Exc-B was dissolved in 100% dimethyl sulfoxide (DMSO) (clear). The experimental concentration of DMSO in DMEM was 0.1%. The control group in DMEM was 0.1% DMSO.

### 4.3. Real-Time PCR Analysis for Cathepsin K and MMP-9 mRNA

The real-time quantitative polymerase chain reaction (qPCR) method used in this study was based on that reported by Livak and Schmittgen (2001) and De Gois et al. (2005) [57,58]. Cell pellets were collected into centrifugation tubes on Days 0, 1, 3, and 6, and total RNA was isolated using TRIzol^®^ RNA Isolation Reagents (Life Technologies, Carlsbad, CA, USA; catalogue no. 15596-026) according to the manufacturer protocol. After centrifugation at 3000× *g* for 8 min at 4 °C, total RNA was obtained and transcribed using the iScript cDNA synthesis kit (Bio-Rad, Hercules, CA, USA). The reactions were setup in duplicate with 0.5 μL of each primer (0.2 μM final concentrations), 25 μL of iQ SYBR Green Supermix (Bio-Rad; 100 mM KCl, 40 mM Tris-HCl, pH 8.4, 0.4 mM of each dNTP, iTaq DNA polymerase, 50 units/mL, 6 mM MgCl_2_, SYBR Green I, 20 nM fluorescein, and stabilizer), and 2.5 μL of template in 50 μL total volumes. The PCR cycle was as follows: 95 °C for 10 min, 40 cycles at 95 °C for 15 s, 60 °C for 1 min, and a melt curve analysis was performed at the end of each experiment to verify that a single product per primer pair was amplified. The amplification and analysis were performed using the CFX96 TouchTM Real-time PCR Detection System (Bio-Rad, Hercules, CA, USA). Results were compared using the relative CT method. The fold increase or decrease was determined relative to a blank control, after normalizing to a housekeeping gene (β-actin) using 2^−∆∆^CT [57,58]. The real-time PCR oligonucleotide primers used for genotyping were as follows: cathepsin K (forward), 5′-AATTGTGACCGTGATAATGTG-3′; cathepsin K (reverse), 5′-GCAGGCGTTGTTCTTATTC-3′; MMP-9 (forward), 5′-GGCGTGTCT GGAGATTCG-3′; MMP-9 (reverse), 5′-ACTGGAAGATGTCGTGTGAG-3′; β-actin (forward), 5′-GCTTCTTTGCAGCTCCTTC-3′; β-actin (reverse), 5′-GACCAGCGC AGCGATATC-3′.

### 4.4. Immunocytochemistry

RAW 264.7 cells were seeded onto slides at a density of 5 × 10^5^ cells and treated with 10 μM Exc-B for 6 days. The modified immunocytochemistry method was performed as previously described [59]. After treatment, RAW 264.7 cells were fixed with 4% paraformaldehyde in phosphate-buffered saline (PBS) for 5 min and then washed three times with TTBS buffer. The cells were blocked with 4% normal Horse serum dissolved in PBS containing 0.01% Triton X-100. After blocking, the RAW 264.7 cells were washed three times with PBS buffer and incubated in a humidified chamber overnight at 4 °C with anti-TRAP polyclonal antibody (1:100; Santa Cruz, CA, USA; catalogue no. sc-28204), or anti-β-actin monoclonal antibody (1:2000; Sigma-Aldrich; catalogue no. A5316-2ML). Cells were washed three times with PBS buffer, blocked as described previously, and then incubated for 1.5 h at 37 °C with Alexa Fluor 488 (green fluorescence) or 594 (red fluorescence)-conjugated secondary antibody. Fluorescence was visualized by microscopic examination (DM 6000B, Leica Inc., Leitz-Park, Wetzlar, Germany) with the SPOT Xplorer Digital camera (Diagnostic Instruments, Inc. Sterling Heights, MI, USA). For quantitative analysis, immunofluorescence data were acquired with 100× magnification, then values of the positive immunoreactive areas were counted using MetaView (Leica, Bensheim, Germany).

### 4.5. Animals

Male Lewis rats (250–280 g) were used for the in vivo experiments, and were obtained from the National Laboratory Animal Centre in Taiwan. The rats were maintained in plexiglass cages in a temperature-controlled (24 ± 1 °C) room under a 12-h light/dark cycle, and given free access to food and water. Each rat was used only once for the experiment. All drug administrations were performed under 2.5% isoflurane anaesthesia. The use of the animals accorded to the Guiding Principles in the Care and Use of Animals of the American Physiology Society and was approved by the institutional animal care and use committee of National Sun Yat-sen University (approved number 10020). Every effort was made to minimize the number of animals used and their suffering.

### 4.6. Adjuvant-Induced Arthritis

The adjuvant-induced arthritic (AIA) rat model in this study was based on that described by Sano et al. and Turull and Queralt [25,60]. In this study, 10 mg/mL heat-killed and lyophilized Mycobacterium butyricum (DIFCO Laboratories, Detroit, MI, USA) was suspended in incomplete Freund’s adjuvant (Sigma-Aldrich) maintained on ice. Rats were then immunized by adjuvant injection of 10 mg/mL M. butyricum in incomplete Freund’s adjuvant. On Day 0, rats were injected intra-dermally at the base of the tail with 0.1 mL of adjuvant, and the development of arthritis was monitored from Day 0 to Day 28. The rats were randomly divided into 5 groups: AIA group (*n* = 7), AIA + Exc-B group (2.5 mg/kg) (*n* = 6), AIA + Exc-B group (5 mg/kg) (*n* = 6), control group (*n* = 6), and Exc-B (5 mg/kg) group (*n* = 6).

### 4.7. Collagen-Induced Arthritis

The collagen-induced arthritic (CIA) rat model used in this study was based on that described by Salvemoni et al. and Brand et al. [61,62]. Bovine type II collagen (MD Biosciences, St. Paul, MN, USA; catalogue no. 804001-lyo) was dissolved (2 mg/mL) in 0.1 M acetic acid at 4 °C overnight. Dissolved CII was stored at −80 °C until used. Animals were immunized with a homogenised emulsion of 1 part CII solution and 1 part incomplete Freund’s adjuvant at 4 °C. On Day 0, randomly selected rats were subcutaneously injected, at the base of the tail, with 0.1 mL of the emulsion, and the development of arthritis was monitored from Day 0 to Day 28. Rats were then randomly divided into 3 groups: CIA group (*n* = 6), CIA + Exc-B group (5 mg/kg) (*n* = 6), and control group (*n* = 6).

### 4.8. Experimental Design

In the AIA + Exc-B or CIA + Exc-B groups, rats received Exc-B 10 times in 2-day intervals between Days 8 and 26. All rats underwent assessments for hindpaw oedema, knee swelling, and body weight, as well as clinical evaluation at Day 0 and before every Exc-B injection between Days 8 and 26. All animals were euthanized by deep anaesthesia with 2.5% isoflurane. The rats were then sacrificed on Day 27 for histopathological analysis and immunochemical staining. All rats were evaluated for arthritic symptoms every 2 days using a macroscopic scoring system, where a score of 0 = no signs of arthritis, 1 = swelling and/or redness of the paw or 1 digit, 2 = two joints involved, 3 = more than two joints involved, 4 = severe arthritis of the entire paw and all digits. The macroscopic score for each rat was calculated by adding the scores of each individual paw. Paw volume was measured using a paw volume meter (Plethysmometer, Singa Inc., Taipei, Taiwan). The change in paw volume was calculated by subtracting the initial paw volume (basal) from the paw volume measured at each time-point [38,63]. Rats received Exc-B (2.5 or 5 mg/kg dissolved in 0.3 mL with 20% DMSO) via subcutaneous injections at each time point.

### 4.9. Histopathological Examination of the Ankle Joint

Rats were sacrificed by perfusion with ice-cold PBS and 4% paraformaldehyde at Day 27 after immunization, and ankle joints were removed and fixed in 10% neutral formalin for 4 days. The ankle joints were decalcified with 12.5% ethylenediaminetetraacetic acid (EDTA) in 10% neutral formalin for 2 weeks and then sectioned in the sagittal plane through the centre of the samples. The specimens were dehydrated with a graded series of increasing alcohol concentrations (Tissue-Tek, Sakura Finetek Japan Co., Ltd., Tokeyo, Japan), embedded in paraffin, and cut into 1-μm sections (Microm, HM340E, Biotechnical Services Inc., North Little Rock, AR, USA) for haematoxylin and eosin and immunohistochemical staining. General and pathological changes in morphology were assessed by microscopic examination using an upright microscope for higher magnification (DM 6000B, Leica Inc., Leitz-Park, Wetzlar, Germany), and a stereomicroscope for lower magnification (APO Z16, Leica Inc., Singapore), with a microscope digital image output system. To quantitatively evaluate the extent of joint destruction in the ankle, the degree of synovial inflammation, bone erosion, and cartilage destruction in each group was scored based on photomicrographs of tissue sections. The degree of synovial inflammation was scored as: 0 = no inflammation, 1 = slight thickening of the synovial cell layer and/or fewer infiltrating cells in the synovial tissue, 2 = mild infiltration of the sub-lining, and 3 = marked-to-severe infiltration. For the degree of bone erosion, 0 = normal, 1 = small areas of resorption, 2 = more numerous areas of resorption, and 3 = full-thickness resorption areas in the bone. For cartilage destruction, 0 = normal, 1 = cartilage surface irregularities, 2 = minor-to-moderate loss of surface cartilage, and 3 = marked cartilage destruction and loss of surface cartilage [64].

### 4.10. Immunohistochemical Staining

Ankle joint specimens were processed for immunohistochemical analysis, as described in previous studies [65,66,67]. Paraffin-embedded ankle joint sections were placed on slides, de-paraffinized with xylene, and dehydrated with a graded series of increasing alcohol concentrations, after which endogenous peroxidase activity was quenched by a 30-min incubation in 0.3% hydrogen peroxide. The antigen was retrieved by enzymatic digestion with 20 mM proteinase K (Sigma-Aldrich) in PBS for 20 min. After washing in ice-cold PBS, slides were incubated in a humidified chamber at 4 °C for 48 h with anti-NFATc1 (1:100; Abcam, Cambridge, MA, USA; catalogue no. ab19027; polyclonal antibody), anti-cathepsin K (1:100; Abcam; catalogue no. ab25916; polyclonal antibody), anti-MMP-2 (1:100, Abcam; catalogue no. ab37150; monoclonal antibody), anti-MMP-9 (1:100; Abcam; catalogue no. ab76003; monoclonal antibody), and anti-CD11b (1:200; Serotec Ltd., Oxford, UK; catalogue no. MCA74GA). The sections were incubated for 90 min with biotinylated anti-rabbit IgG (Vector Laboratories, Burlingame, CA, USA) and diluted 1:200 in 1% bovine serum albumin (BSA) in PBS. Sections were then immunohistochemically labelled with the avidin-biotin complex technique using an ABC kit (Vectastain ABC kit, Vector Laboratories Inc., Las Vegas, NV, USA). Finally, the sections were treated with 3,3′-diaminobenzidine tetrahydrochloride (DAB; Peroxidase substrate kit, Vector Laboratories Inc., Las Vegas, NV, USA) for 1–5 min. All slides for immunohistochemistry were analysed under a light microscope (DM 6000B, Leica Inc., Leitz-Park, Wetzlar, Germany) with a microscope digital image output system (SPOT idea 5.0 Mp Colour Digital Camera, Diagnostic Instruments Inc., Sterling Heights, MI, USA). All slides for immunochemical staining were analysed using an upright microscope in combination with a digital image output system. The total number of immunoreactive positive cells was determined with 200× magnification in six fields under a light microscope, and the results were averaged.

### 4.11. Preparation of Synovial Tissue for Western Blot Analysis

Rats were sacrificed at Day 27 after immunization, and the knee synovial tissues were collected for Western blot analysis from the control groups, AIA group, AIA + Exc-B group (2.5 mg/kg), AIA + Exc-B group (5 mg/kg), CIA group, and CIA + Exc-B group (5 mg/kg). The synovial tissues were collected and washed with PBS and homogenized in lysis buffer using a polytron homogenizer (Precellys^®^24, tissue homogenizer, Bertin Technologies, Aix-en-Provence, France). The samples were then ultracentrifuged at 65,000 rpm for 1 h at 4 °C (Beckman Coulter Optima TLX-120 Ultracentrifuge, Beckman Coulter Inc., Brea, CA, USA), and the supernatant was collected and assessed using a DC protein assay kit (Bio-Rad). Protein samples were then used for Western blot analysis. We electrophoresed the protein in tricine SDS-polyacrylamide (10%) gel at 80 V for 180 min. Proteins were transferred to polyvinylidene difluoride (PVDF) membranes (Immobilon-P; pore size 0.45 μM; Millipore, Bedford, MA, USA) at 135 mA for 16–18 h at 4 °C in transfer buffer (50 mM Tris–HCl, 380 mM glycine, 1% SDS, 20% methanol). Membranes were blocked for 60 min at room temperature with 5% non-fat dry milk in Tris-buffered saline with Tween 20 (TTBS; 0.1% Tween 20, 20 mM Tris–HCl, pH 7.4, 137 mM NaCl) and then incubated overnight at 4 °C with primary antibodies against anti-HO-1 (1:1000; Enzo Life Sciences, Farmingdale, NY, USA; catalogue no. ADI-SPA-895), anti-HMGB-1 (1:1000; Cell Signaling Technology Inc., Danvers, MA, USA; catalogue no. 3935), anti-p-ERK (1:1000; Cell Signaling Technology Inc.; catalogue no. 9102), anti-ERK (1:1000; Gene Tex Inc., Irvine, CA, USA; catalogue no. gtx627408), anti-p-JNK (1:1000; Cell Signaling Technology Inc.; catalogue no. 4668), anti-JNK (1:1000; Cell Signaling Technology Inc.; catalogue no. 9252), anti-p-P38 (1:1000; Cell Signaling Technology Inc.; catalogue no. 4511), anti-P38 (1:1000; Cell Signaling Technology Inc.; catalogue no. 9212), and GAPDH (1:1000; Gene Tex Inc.; catalogue no. gtx627408). The immunoreactive bands were visualized by enhanced chemiluminescence (Millipore, Billerica, MA, USA) and the Biochemi Imaging System, and relative densitometric quantification was performed using LabWorks 6.2 software (UVP, Upland, CA, USA).

### 4.12. Measurements of Serum IL-17A and M-CSF Levels

Levels of the pro-inflammatory cytokines IL-17A and M-CSF in blood serum of the rats were measured at Day 28 after immunization by using Bio-Plex kits (Bio-Rad, Hercules, CA, USA), in accordance with the manufacturer’s recommendations. Samples were thawed and centrifuged at 10,000× *g* for 10 min at 4 °C. Briefly, 50 μL of the sample was incubated with antibody-coupled beads for 60 min at 25 °C. After washing three times to remove unbound materials, the beads were incubated with biotinylated detection antibodies for 30 min at room temperature. After washing away the unbound biotinylated antibodies with three washes, the beads were incubated with streptavidin-PE for 10 min at room temperature. Following removal of excess streptavidin-PE with three washes, the beads were re-suspended in 125 μL of assay buffer. Finally, beads were read using the Bio-Plex suspension array system, and the data were analysed using Bio-Plex Manager software (Bio-Rad, Hercules, CA, USA) [68].

### 4.13. Statistical Analysis

All data are presented as mean ± SEM. The data for foot oedema, mean arthritis score and weight gain were analysed by Kruskal–Wallis One Way Analysis of Variance analysis along with normality test (*p* < 0.05). Other data were analysed using one-way analysis of variance (ANOVA) followed by the Student–Newman–Keuls post hoc test (SigmaPlot 11.0 for Windows, Systat Software, Inc. San Jose, CA, USA). Differences resulting in *P* values less than 0.05 were considered significant.

## 5. Conclusions

Our previous study revealed that Exc-B had anti-inflammatory and analgesic effects in a carrageenan-induced oedema model. In this study, we therefore continued our evaluation of Exc-B with cultured-type gorgonian *B. excavatum*. The results showed that Exc-B could significantly inhibit osteoclast differentiation on LPS-induced multinucleated cell formation by downregulating MMP-9 and cathepsin K gene expression and TRAP protein expression. Administration of Exc-B (2.5 or 5 mg/kg) also significantly inhibited foot oedema in AIA, as well as arthritis score, and body weight loss. Similar results were observed with the CIA model. Moreover, histopathological and immunohistochemical examinations further demonstrated that systemic injection of Exc-B attenuated histological features of AIA and CIA in the ankle joint by downregulating the number of TRAP-positive multinucleated cells. Cathepsin K, MMP-2, and MMP-9 protein expressions were also reduced with downregulation of the transcription factor NFATc1 protein expression, after Exc-B treatment. Moreover, we determined the effect of Exc-B on the MAPK and HO-1/HMGB-1 pathways in synovial tissue via Western blotting to detect the level of IL-17A and M-CSF in the serum. The results showed that Exc-B significantly inhibited MAPK, HMGB-1, IL-17A, and M-CSF and up-regulated HO-1 protein in AIA and CIA rats. We conclude that Exc-B could reduce osteoclastogenesis via downregulation of the inflammatory factors IL-17A and M-CSF to influence the MAPK and HO-1/HMGB-1 pathways in AIA and CIA rats.

## Supplementary Materials

The following are available online at www.mdpi.com/1660-3397/15/1/9/s1. Figure S1: Effect of Exc-B on LPS induced Osteoclast like cell model.
