# Supplementary material for: Excavatolide B Attenuates Rheumatoid Arthritis through the Inhibition of Osteoclastogenesis
Source: Mar Drugs. 2017 Jan 6;15(1):9. doi: 10.3390/md15010009 (PMC5295229; doi:10.3390/md15010009)
Supplement: Supplementary file 1 [file marinedrugs-15-00009-s001.docx]

Supplementary Materials: Excavatolide B
Attenuates Rheumatoid Arthritis through
the Inhibition of Osteoclastogenesis

Yen-You Lin, Yen-Hsuan Jean, Hsin-Pai Lee, Sung-Chun Lin, Chieh-Yu Pan, Wu-Fu Chen,
Shu-Fen Wu, Jui-Hsin Su, Kuan-Hao Tsui, Jyh-Horng Sheu, Ping-Jyun Sung and Zhi-Hong Wen

| 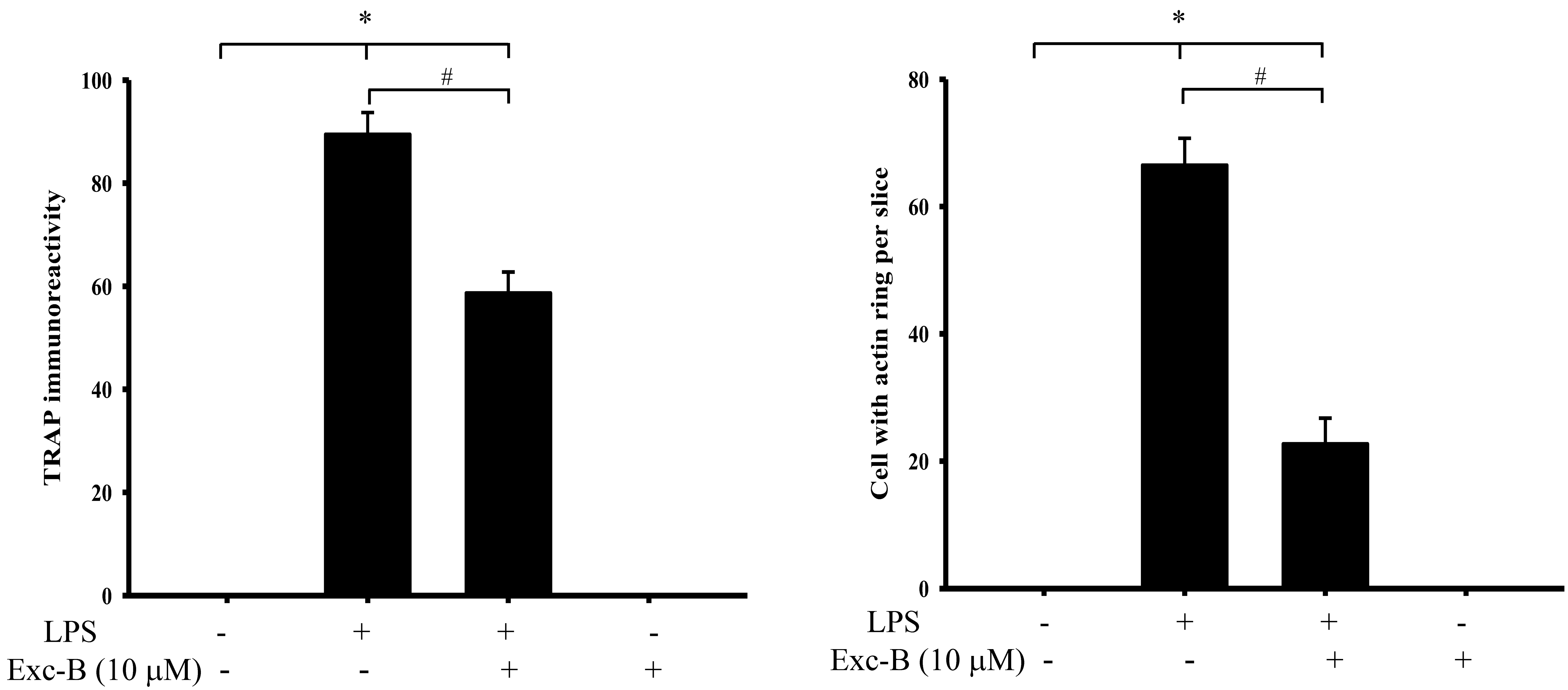 | |
| --- | --- |
| (**A**) | (**B**) |

**Figure S1.** Effect of Exc-B on LPS induced Osteoclast like cell model. (**A**) The quantification of TRAP immunoreactivity in LPS-stimulated osteoclast-like cells; (**B**) The number of actin rings in LPS-stimulated osteoclast-like cells. The data are representative of three independent experiments. Values reflect the mean ± SEM for each group. The data were analyzed by one-way analysis of variance (ANOVA) followed by the Student-Newman-Keuls post hoc test. * *p* < 0.05 compared with the control group; # *p* < 0.05 compared with the LPS treatment alone group.
